# Development of a novel Guinea Pig model producing transgenerational endothelial transcriptional changes driven by maternal food restriction and a second metabolic insult of high fat diet

**DOI:** 10.3389/fphys.2023.1266444

**Published:** 2023-10-24

**Authors:** Hillary H. Le, Matthew W. Hagen, Samantha Louey, Hagai Tavori, Kent L. Thornburg, George D. Giraud, Monica T. Hinds, Anthony P. Barnes

**Affiliations:** ^1^ Department of Biomedical Engineering, Oregon Health and Science University, Portland, OR, United States; ^2^ Center for Developmental Health, Portland, OR, United States; ^3^ Knight Cardiovascular Institute, Portland, OR, United States; ^4^ Department of Chemical Physiology and Biochemistry, Oregon Health and Science University, Portland, OR, United States; ^5^ VA Portland Health Care System, Portland, OR, United States

**Keywords:** maternal diet, transgenerational, endothelium, transcriptome, guinea pig (*Cavia porcellus*)

## Abstract

Developmental programming of chronic adverse cardiovascular health outcomes has been studied both using numerous human populations and an array of animal models. However, the mechanisms that produce transgenerational effects have been difficult to study due to a lack of developmentally relevant models. As such, how increased disease risk is carried to the second generation has been poorly studied. We hypothesized that the endothelium which mediates many acute and chronic vascular inflammatory responses is a key player in these effects, and epidemiological studies implicate transgenerational nutritional effects on endothelial health. To study the mutigenerational effects of maternal undernutrition on offspring endothelial health, we developed a model of transgenerational nutritional stress in guinea pigs, a translationally relevant precocial species with a relatively short lifespan. First- and second-generation offspring were subjected to a high fat diet in adolescence to exacerbate negative cardiovascular health. To assess transcriptional changes, we performed bulk RNA-sequencing in carotid artery endothelial cells, with groups stratified as prenatal control or food restricted, and postnatal control or high fat diet. We detected statistically significant gene alterations for each dietary permutation, some of which were unique to treatments and other transcriptional signatures shared by multiple or all conditions. These findings highlight a core group of genes altered by high fat diet that is shared by all cohorts and a divergence of transgenerational effects between the prenatal *ad libitum* and dietary restriction groups. This study establishes the groundwork for this model to be used to better understand the interplay of prenatal stress and genetic reprogramming.

## Introduction

The developmental origins of disease (DOHaD) is a well-established concept in which sub-optimal intrauterine conditions increase the risk of chronic cardiovascular and metabolic diseases (Heindel et al., 2015; Hoffman et al., 2017; Justulin et al., 2023). Adaptations made by the fetus in response to these adverse conditions may initially be subtle, but persist and amplify postnatally leading to organ and systems dysfunction. A secondary challenge or “second hit” may exacerbate these dysfunctions, accelerating the path to disease; indeed, several animal models indicate that the negative effects of a prenatal stress are not revealed until a secondary stressor is applied.

Increasing evidence indicates adverse developmental conditions may also produce multigenerational effects (Heard and Martienssen, 2014; Hagemann et al., 2021), but the challenge in investigating the mechanisms of these effects lies in a lack of suitable models. Life-course studies are time consuming, and choice of a suitable developmental model is important for translational value to human health (Chavatte-Palmer et al., 2016; Dickinson et al., 2016; Hammer et al., 2023). We have chosen to establish this transgenerational model in guinea pigs (*Cavia porcellus*) because of their similarities to humans including a hemochorial placenta, precocial organ development timelines, and plasma lipid profiles (Davies et al., 1961; Dwyer et al., 1992; Noonan, 1994; Cos et al., 2001; Fernandez, 2001; Carter, 2007).

Guinea pig offspring have been shown to be vulnerable to the effects of suboptimal intrauterine conditions, whether the stressor is disruption of maternal uterine artery blood flow (by acute ligation, ablation, or gradual restriction), maternal undernutrition, or litter size (Garris, 1983; Garris, 1984; Jones et al., 1984; Lafeber et al., 1984; Jansson et al., 1986; Carter and Detmer, 1990; Jansson and Persson, 1990; Jones et al., 1990; Widmark et al., 1990; Detmer et al., 1991; Widmark et al., 1991; Detmer et al., 1992; Dwyer et al., 1992; Persson and Jansson, 1992; Carter, 1993; Turner, 1997; Roberts et al., 2001; Briscoe et al., 2004; Carter et al., 2005; Cross and Mickelson, 2006; Turner and Trudinger, 2009; Thompson et al., 2011; Sarr et al., 2014; Thompson et al., 2014; Herrera et al., 2016; Horton et al., 2016; Sarr et al., 2016; Morrison et al., 2018). Uterine artery ablation may increase the likelihood of low birth weight (LBW) offspring (Sarr et al., 2014; Sarr et al., 2019) but when pups are born spontaneously, it is not clear whether pups were from the ablated uterine horn or the contralateral control horn; in studies like these, an arbitrary designation of LBW <25th percentile may be set to define “prenatally stressed” offspring separate to normal birth weight (“unstressed”) offspring. A subset of these designated LBW offspring may be naturally occurring (Horton et al., 2016), which mechanistically could have different programming effects from normally-growing offspring whose growth trajectory has been derailed in mid-to-late gestation. The combination of these two types of LBW offspring may explain some of the small effects reported by designating offspring by this method (Thompson et al., 2014; Horton et al., 2016); likewise some offspring from the ablated uterine horn may be born with “normal” birth weights, potentially having a different adaptive growth capacity than their littermates to maintain somatic growth, although no comment can be made at a tissue or organ level (Owens et al., 1987; Reynolds et al., 2005; Roberts et al., 2012). More recently, gradual constriction of the bilateral uterine arteries has been shown to produce an asymmetrical intrauterine growth restriction (IUGR) phenotype (with brain sparing and abdominal wasting) (Herrera et al., 2016) that may reflect a better measure of vulnerability to DOHaD than a mere reduction in birth weight. Thus, birth weight may have limited value in its use as a surrogate for sub-optimal intrauterine conditions.

Maternal undernutrition in guinea pigs leads to altered cholesterol and glucose homeostasis in offspring (Kind et al., 1999; Kind et al., 2003; Kind et al., 2005; Torres-Gonzalez et al., 2008; Nguyen et al., 2010), indicating this prenatal insult has long-term metabolic consequences in line with developmental programming, and unlike the vascular ablation model, all pups within a litter are known to have been subject to the same maternal insult, regardless of birth weight. Guinea pigs are also sensitive to high fat diets, with outcomes including altered cholesterol levels, atherosclerotic lesions, hepatic steatosis, and vascular dysfunction and stiffness (Weber and Tosi, 1971; Yount and McNamara, 1991; Fernandez et al., 1995; Fernandez et al., 1996; Sharman et al., 2008; Krieglstein et al., 2010; Rice et al., 2010; Rice et al., 2012; Ye et al., 2013; Thompson et al., 2014; Sarr et al., 2019). However, studies have not assessed the effects of maternal feed restriction that does not result in low birth weight nor the compounded effects of the secondary impact of high fat diet (HFD). For these reasons, we sought to develop a transgenerational model using guinea pigs to assess these questions by analyzing transcriptome of carotid endothelial cells.

Prenatal stress is known to blunt endothelium-dependent responsiveness (Torrens et al., 2009; Torrens et al., 2012; Thompson et al., 2014). Endothelial cells are directly involved in many cardiovascular diseases and inflammatory processes including atherosclerosis. Many studies have demonstrated a link between cardiovascular disease (CVD) and nutritional restriction with low birth weight and without low birth weight (Dodic et al., 1998; Dodic et al., 2002). A singular insult of food restriction has been studied in various animal models including guinea pigs. (Langley-Evans et al., 1996; Hawkins et al., 2000; Edwards and McMillen, 2001; Nevin et al., 2018). The combination of a moderate food restriction *in utero* with a second challenge of HFD later in life is understudied in animal models. We hypothesized endothelial dysfunction may not just underlie developmental programming in offspring but create epigenetic changes that impact the subsequent generations (DeRuiter et al., 2008; Gluckman et al., 2008; Ambrosini et al., 2020). Therefore, we chose to analyze the transcriptome of endothelial cells to identify genes that may be associated with increased risk of cardiovascular disease development.

## Methods

### Animal model

**FIGURE 1:**
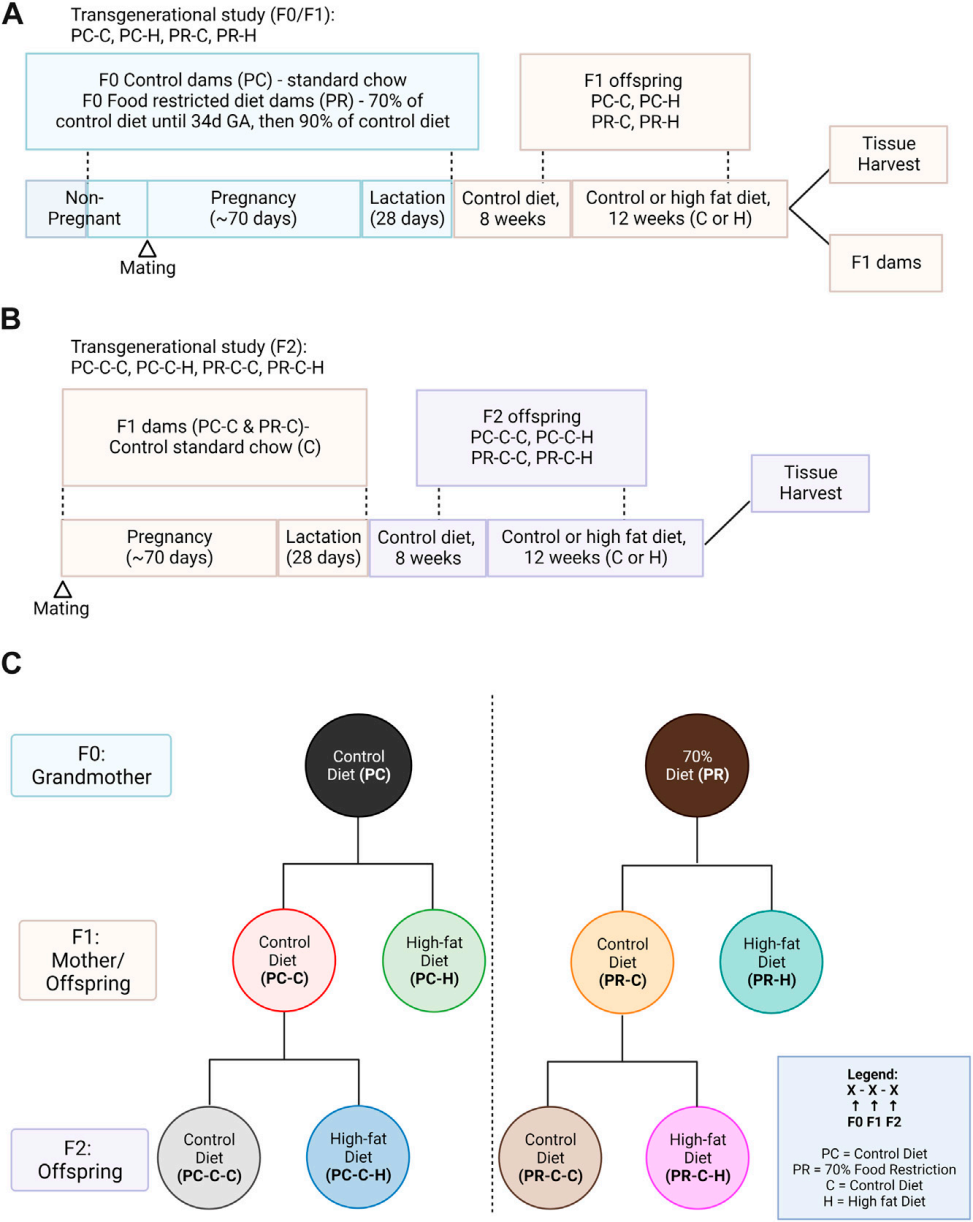
Study design. **(A)** F0 guinea pigs were fed a control *ad libitum* (PC) diet (*n* = 4) or restricted (PR) diet (*n* = 4) from the periconceptional period through lactation. F1 offspring were fed a control *ad libitum* diet until 8 weeks of age and which offspring received either an *ad libitum* high fat (−H) chow or remained on control *ad libitum* (−C) chow for 12 weeks. **(B)** A subset of PC-C and PR-C offspring were bred to determine the transgenerational effects of prenatal diet on the second generation; these F2 offspring were similarly exposed to the 12-week high fat diet from 8 weeks of age. **(C)** Overview of study group notation. Created with Biorender.com.

The study protocol approved by the Institutional Animal Care and Use Committee of Oregon Health and Science University (OHSU, IP00000012). The model design is illustrated in Figure 1.

### F0 prenatal diet

Two adult male (550–600 g) and 8 nulliparous female (400–450 g) Hartley Guinea Pigs were purchased from Charles River Laboratories and housed in individual cages. All animals were weighed three times per week. Purchased animals were fed a standard, control chow (LabDiets 5025, 13% energy from fat, 27% from protein, 60% from carbohydrate) *ad libitum* for 3 weeks to acclimate and weight and food intake were recorded daily. Following this acclimatization period, half of the female guinea pigs received 70% of the average (per kg body weight) daily *ad libitum* diet (PR; PrenatalFood Restriction) for 3 weeks; the remainder of the females (PC; Prenatal *Ad libitum* Control), and all males received 100% of the average (per kg body weight) daily *ad libitum* diet. Male and female guinea pigs were mated overnight, and mating confirmed by the presence of a vaginal plug. Control guinea pigs remained on their designated diet for the duration of gestation (∼70 days) and throughout lactation (∼28 d days). Food-restricted dams remained on 70% of *ad libitum* diet until 34 dGA (days gestational age, ∼mid gestation), after which they were placed on 90% of *ad libitum* diet until the end of lactation; this increase in feed is to prevent weight loss during the latter half of pregnancy (Kind et al., 1999; Roberts et al., 2001; Kind et al., 2003).

### F1 generation offspring

Offspring were born spontaneously and litter size was culled to 3 pups per dam. Offspring were housed with their mother until weaning at 28 days. Tissues from pups culled at birth were preserved for future studies; pup selection for culling was pseudo-random in order to maintain equal numbers of male and female offspring in each study group, and to maintain equal mean pup weights between culled and unculled groups. After weaning, all offspring were fed a control diet until at least 8 weeks of age.

A subset of female F1 offspring were used as breeders to produce F2 offspring, and remained on an *ad libitum* control chow diet for the remainder of the study, including during pregnancy. F1 breeders were mated with newly-purchased unrelated adult male Hartley Guinea Pigs purchased from Charles River Laboratories, as described above.

At 8 weeks of age, half of remaining non-breeding F1 offspring were fed *ad libitum* control chow (PC-C or PR-C) while other half were placed on an *ad libitum* high fat chow (PC-H or PR-H) for 12 weeks; food intake was monitored daily, and animals were weighed 3 times per week. The high fat chow was based on the standard lab chow LabDiet 5025, supplemented with 0.25% cholesterol, 15% fat from pork fat, and 2.5% fructose (38% energy from fat, 20% from protein, 42% from carbohydrate). Offspring diets were pseudo-randomly assigned to avoid siblings receiving the same treatment.

### F2 generation offspring

Offspring were born spontaneously and treated as described above for the non-breeding F1 offspring. At 8 weeks of age, offspring received either *ad libitum* control chow (PC-C-C or PR-C-C) or *ad libitum* high fat chow (PC-C-H or PR-C-H).

### Serum cholesterol and triglycerides

Blood samples (0.3–0.5 mL) were collected from the tarsal or saphenous vein from non-fasted animals for standard lipid assays at the start (8 weeks of age) and after 12 weeks of the high fat diet challenge (20 weeks of age). Serum was isolated by centrifugation 1000 rpm × 10 min, and samples were stored at −80C until analysis. Serum samples were analyzed using liquid reagents for cholesterol or triglycerides (both from Pointe Scientific, Canton, Michigan). Absorbance was measured at 490 nm for cholesterol and at 540 nm for triglycerides using a microplate reader (SpectraMax iD3, Molecular Devices, San Jose, California) using cholesterol and glycerol standards.

### Endothelial cell isolation

At 20 weeks of age, 3 animals each from PC-C, PC-H, PR-C, PR-H, PC-C-C, PC-C-H, PR-C-C, PR-C-H were euthanized with intravenous overdose of commercial barbituate (Somnasol, 390 mg/mL pentobarbital sodium). A carotid artery was rapidly cannulated *in situ*. The vessel was flushed with 1X PBS (Gibco) to remove blood, followed by 100uL of TRIzol Reagent (Invitrogen) to collect endothelial cell contents. RNA was isolated using RNeasy Micro kit (Qiagen) and quality was assessed using Agilent 2,100 Bioanalyzer with RNA 6000 Pico chip by the OHSU Gene Profiling Shared Resource. All samples had an RNA Integrity Number (RIN) score of above 5.0. Samples were stored at −80C until sequencing.

### RNA sequencing and analysis

Short-read sequencing assays were performed by the OHSU Massively Parallel Sequencing Shared Resource. Raw data was checked for quality using FastQC, trimmed using Trimmomatic, aligned using STAR, and analyzed for differential gene expression using DESeq2 in R (Love et al., 2014). The raw and processed RNA-seq dataset used and analyzed in this current study are publicly available through NCBI Gene Expression Omnibus (GEO) via accession series GSE244302.

### Statistical analysis

Statistical analysis was performed using R (R foundation for statistical computing, Vienna, Austria). All data were checked for normality using a Shapiro-Wilk test and equality of variance using Levene’s Test. Normally distributed data are displayed as mean ± standard deviation (SD) and analyzed with Student’s t-test (two groups) or analysis of variance (more than two groups) with Tukey’s *post hoc* where appropriate. Non-normally distributed data were analyzed using Wilcoxon signed-rank test (two groups) or Kruskal–Wallis Test (more than two groups) with Dunn’s *post hoc* with Bonferroni correction where appropriate. Data are considered statistically significant when *p* < 0.05.

### Pathway analysis and venn diagrams

Pathway analysis and graphing were performed using STRING database algorithm (Hong et al., 2014). Venn diagrams were generated using the BioVenn algorithm (Hulsen et al., 2008) for comparisons of less than four conditions and the DeepVenn (Hulsen, 2022) for those exceeding three conditions.

## Results

### Prenatal food restriction effects

The F0 dams in the food restricted treatment group were similar in weight to control diet dams (*p* = 0.370). Food restricted diets had significantly less total kilocalorie/kg/day intake including kilocalorie/kg/day from fat, protein and carbohydrates compared to control animals (*p* < 0.05). However, the treatment did not significantly alter the dams’ body weight during pregnancy or lactation (Table 1; Supplementary Figure S1). Lipid analysis of F0 dams’ serum showed unaltered total cholesterol, but triglycerides were increased in food restricted dams (Supplementary Figure S2). There were no differences in age or weight at conception, pregnancy rate or offspring gestational age, litter size or surviving young (Table 2). For the PC and PR F1 dams, there were no differences in age or weight at conception, weight gain during pregnancy or lactation, pregnancy rate or offspring gestational age, litter size or surviving F2 offspring (Table 2; Supplementary Figure S1).

**TABLE 1:** F0 Dams’ kilocalorie/kg/day intake and body weight (g). Food restricted diets had significantly less total kilocalorie/kg/day intake including kilocalorie/kg/day from fat, protein and carbohydrates compared to control animals (*p* < 0.05). However, the treatment did not significantly alter the dams’ body weight. For both control and food restricted diet groups, *n* = 4. Pre-treatment and treatment measurements spanned 3 weeks. All data are displayed as mean ± SD and were checked for normality using a Shapiro-Wilk test and homogeneity of variance and using Levene’s Test and quantile-quantile plots.

	Body weight (g)		Total kcal/kg/day		Fat kcal/kg/day		Protein kcal/kg/day		Carbohydrate kcal/kg/day	
Treatment	Baseline	After Diet	Baseline	After Diet	Baseline	After Diet	Baseline	After Diet	Baseline	After Diet
Control	612 ± 45	693 ± 71	232 ± 67	186 ± 29	31 ± 9	25 ± 4	61± 18	50 ± 8	139 ± 40	112 ± 17
Restricted	617 ± 28	652 ± 46	219 ± 38	145 ± 6***	29 ± 5	19 ± 1***	58 ± 10	39 ± 2***	132 ± 23	87 ± 4***

**TABLE 2:** F0 and F1 dams’ pregnancy data. For F0 mothers, *n* = 4 in prenatal control diet and *n* = 4 in prenatal food restricted diet. For F1 mothers, *n* = 2 in prenatal control diet and *n* = 4 in prenatal food restricted diet. All data were checked for normality using a Shapiro-Wilk test and equality of variance using Levene’s Test. Data that were normally distributed are displayed as mean ± SD and were analyzed using Student’s T-test. † denotes data that are not normally distributed which are displayed as median ± IQR and were analyzed using Wilcoxon signed-rank test.

	Control	Food Restricted	*p*-value
**F0 Dams**			
Age at conception (days old)	141 ± 19	148 ± 19	0.6
Weight at mating (g)	706 ± 126	726 ± 126	0.8
Pregnancy rate^†^ (%)	75	75	0.9
Gestational age at birth^†^ (days old)	69 ± 0	70 ± 1	0.4
Litter size^†^	4 ± 1	3 ± 0	0.6
Surviving young^†^ (%)	100	100	1.0
**F1 Dams**			
Age at conception (days old)	146 ± 26	128 ± 8	0.5
Weight at mating (g)	682 ± 4	730 ± 106	0.8
Pregnancy rate (%)	100	100	1.0
Gestational age at birth (days old)	68 ± 0	67 ± 1	0.5
Litter size	4 ± 1	4 ± 1	1.0
Surviving young (%)	100	100	0.5

### Postnatal growth rates

**FIGURE 2:**
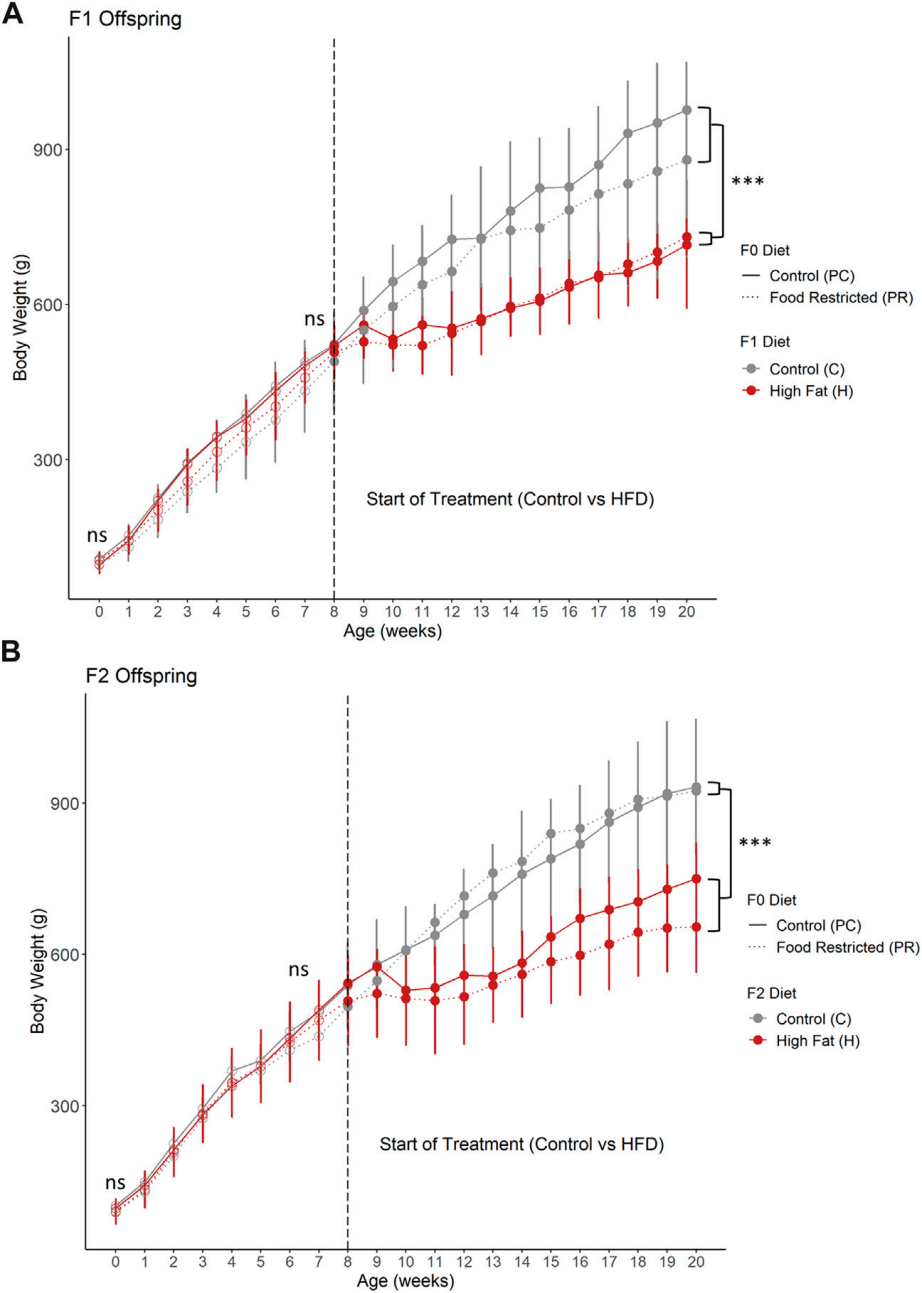
**(A)** F1 and **(B)** F2 offspring body weight from birth to 20 weeks of age. **(A)** F1 offspring from prenatal food restriction did not significantly differ in body weight at birth or at 8 weeks of age. Following 12 weeks of high fat diet, body weight was significantly less than control diet offspring. Additionally, **(B)** F2 offspring also did not significantly differ in body weight at birth or at 8 weeks of age. After 12 weeks of high fat diet, body weight was significantly less than control diet animals. For F1 prenatal control, postnatal control (PC, *n* = 3 for postnatal control diet (PC-C) and *n* = 4 for high fat postnatal diet (PC-H). For F1 prenatal food restricted diet offspring, *n* = 3 for postnatal control diet (PR-C) and *n* = 4 for high fat postnatal diet (PR-H). For F2 prenatal control diet offspring, *n* = 3 for both postnatal control (PC-C-C) and high fat diet (PC-C-H). For F2 prenatal food restricted diet offspring, *n* = 3 for postnatal control diet (PR-C-C) and *n* = 7 for high fat postnatal diet (PR-C-H). All data points are displayed as mean ± SD and were checked for normality using a Shapiro-Wilk test and quantile-quantile plat as well as homogeneity of variance using Levene’s Test. Birth weight, weight at start of treatment (8 weeks of age), and weight at end of study (20 weeks of age) were analyzed using 2 way ANOVA. Tukey’s post-hoc test was used when significant. ***p < 0.001. ns = not significant.

The offspring from prenatal food restriction in both F1 (Figure 2A) and F2 (Figure 2B) generations did not differ in birth weight or weight at 8 weeks of age. After 12 weeks of high-fat diet, both F1 and F2 offspring, high fat diet animals weighed significantly less than control diet animals (*p* < 0.005). Both PC and PR F1 offspring had transient weight loss following the initiation of the HFD with lower weight gain during the HFD period such that by 20 weeks old, the HFD animals weighed significantly less than control-diet fed animals (Figure 2A, *p* = 0.008, 2-way ANOVA). Despite the high fat animals weighing less, their total kilocalories/kg/day was comparable to control animals but the calories from fat was significantly higher (Supplemental Table S1). F2 offspring took longer to acclimate to the HFD diet, with 5 weeks of negligible weight gain, followed by continued low weight gain (Figure 2B, *p* = 0.0005). This was supported by decreased total kilocalories/kg/day in the F2 HFD group in which calories consumed from fat, protein and carbohydrate sources were similar to control animals (Supplemental Table S1).

### High fat diet treatment

**FIGURE 3:**
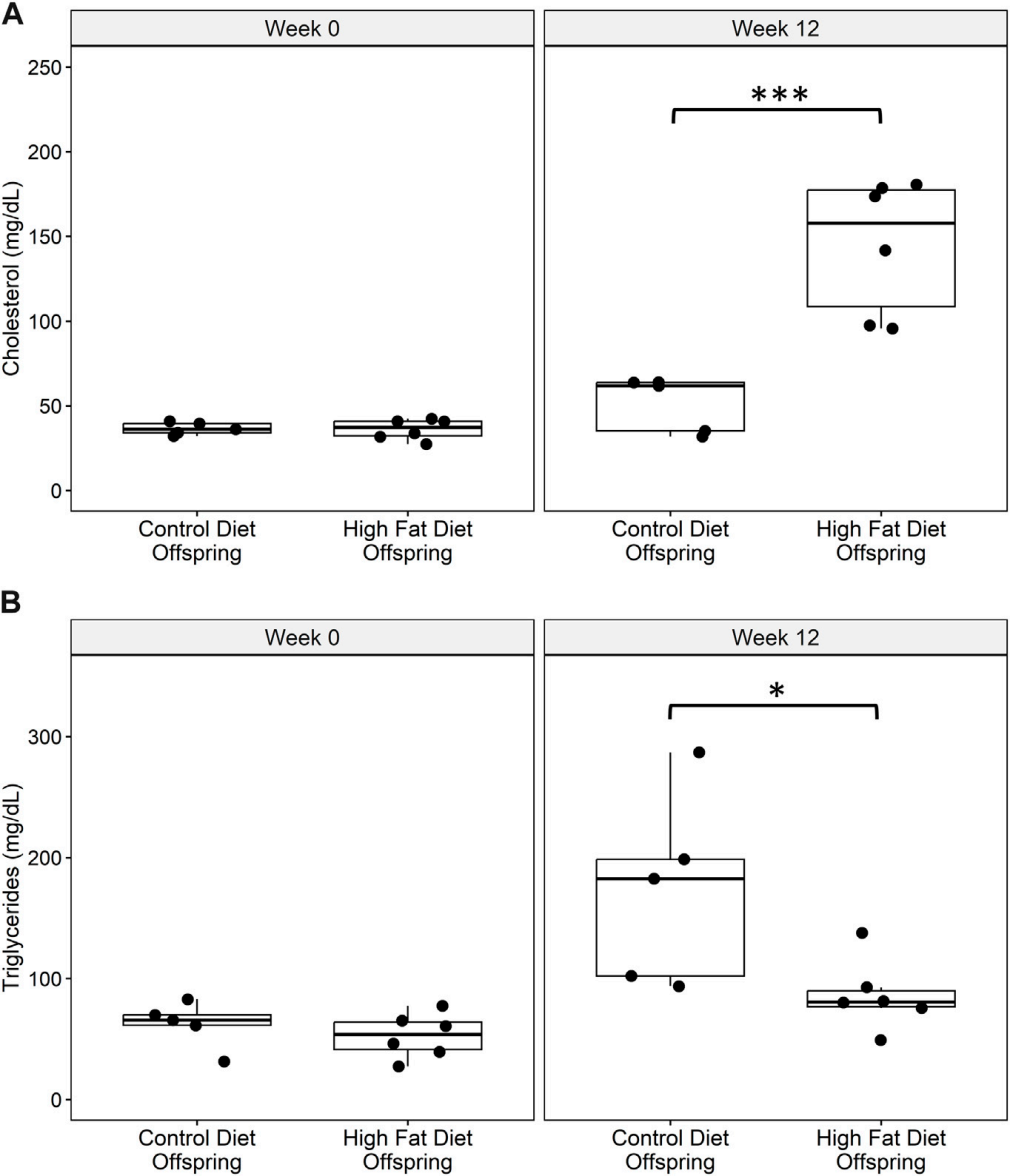
**(A)** Total serum cholesterol and **(B)** serum triglyceride levels at baseline and at week 12 of the high fat diet challenge for control diet offspring (*n* = 5) and high fat diet offspring (*n* = 6). All data points are displayed and analyzed using Wilcoxon Test. Data were checked for normality using a Shapiro-Wilk test and quantile-quantile plot as well as equality of variance using Levene’s Test. *p < 0.05, ***p < 0.005.

There are no differences in total cholesterol or triglyceride levels between groups at start of treatment. After 3 weeks on treatment, HFD animals had significantly higher total cholesterol levels than control animals (*p* = 0.005), while triglycerides did not increase. Offspring placed on the HFD challenge had increased levels of total serum cholesterol after 12 weeks in comparison to offspring fed a control chow diet (Figure 3A). HFD offspring had lower serum triglycerides than controls at the conclusion of the 12-week challenge period (Figure 3B).

### Transcriptional changes in carotid endothelial cells

**FIGURE 4:**
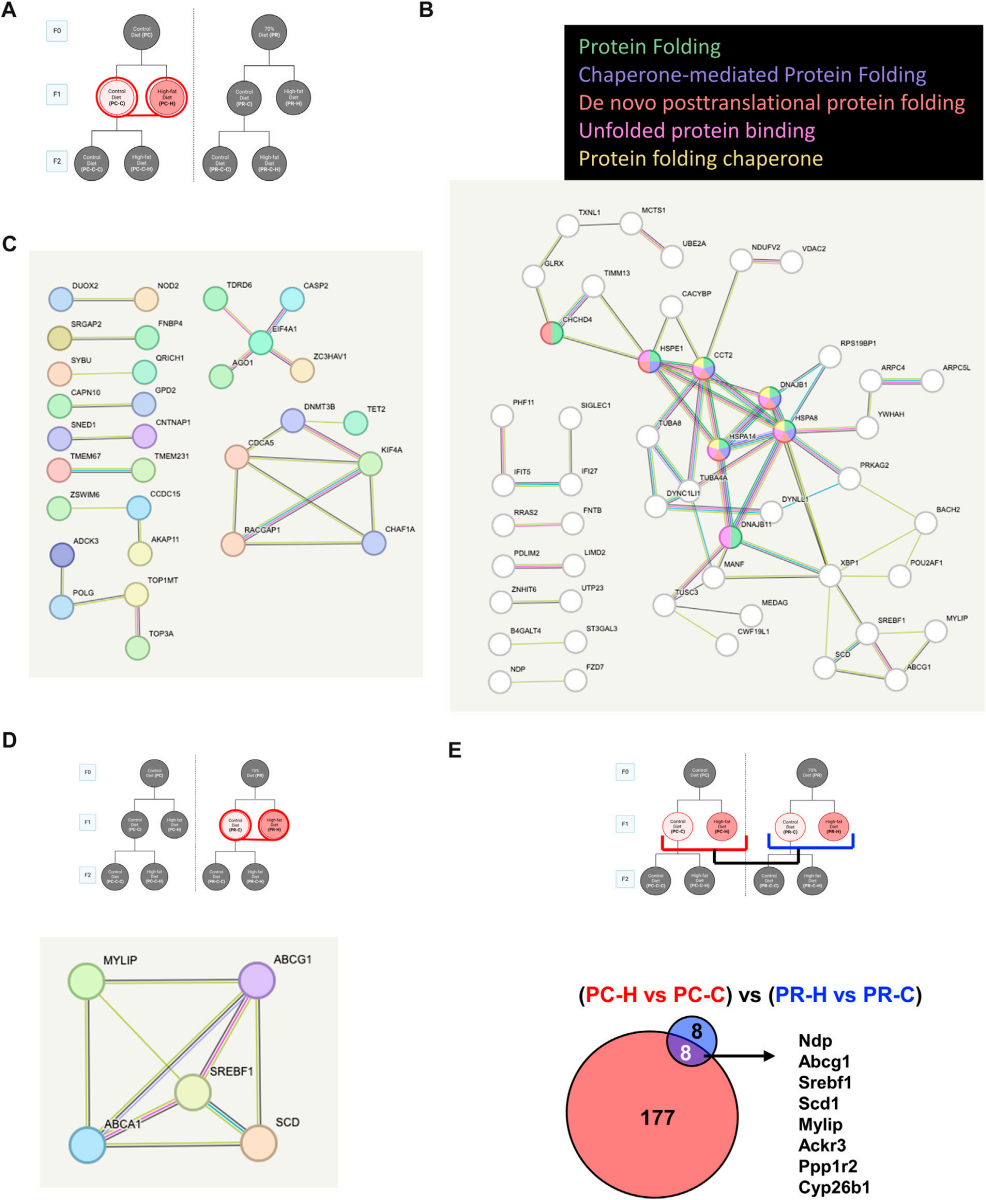
Control diet comparisons. **(A)** Schematic of comparison for PC-C vs PC-H which revealed 185 differentially expressed genes (DEGs). STRING pathway analysis of **(B)** upregulated DEGs that are associated with specific Gene Ontology (GO) terms while **(C)** downregulated DEGs lacked specific GO term assignments. **(D)** The F1 generation with high fat diet from restricted diet F0 arm (PR-C vs PR-H) exhibited fewer DEGs (16 genes) some of which form a network of lipid metabolism-related genes. **(E)** Comparing overlap of PC-C vs PC-H (185 DEGs) and PR-C vs PR-H (16 DEGs) of F1 high fat diet offspring from control and restricted dams have 8 DEGs in common.

All samples except one from the PC-C-H condition were found to be good quality and moved forward to differential expression analysis. Initial testing for vascular markers confirmed our enrichment of endothelial cell mRNA in our samples (Supplementary Figure S3). Pair-wise comparisons of conditions indicate the response of gene networks in a subset of cohorts. In the case of the control F0 diet (PC) F1 generation (Figure 4A), several up- and downregulated genes (Figures 4B, C, respectively) could be assigned to interrelated networks. Those genes exhibiting increased expression were associated with specific Gene Ontology (GO) terms related to protein folding (Figure 4B), while genes with decreased expression lacked specific GO term assignments but did have several clear protein networks (Figure 4C). As noted above, the F1 generation from restricted diet F0 arm (PR) exhibited many fewer differential genes (16 genes) some of which form a network of lipid metabolism-related genes (Figure 4D). Interestingly, half of the gene differentially expressed in the dietary restriction arm of the F1 generation are also observed to be altered in the F1 generation of the control arm (Figure 4E).

**FIGURE 5:**
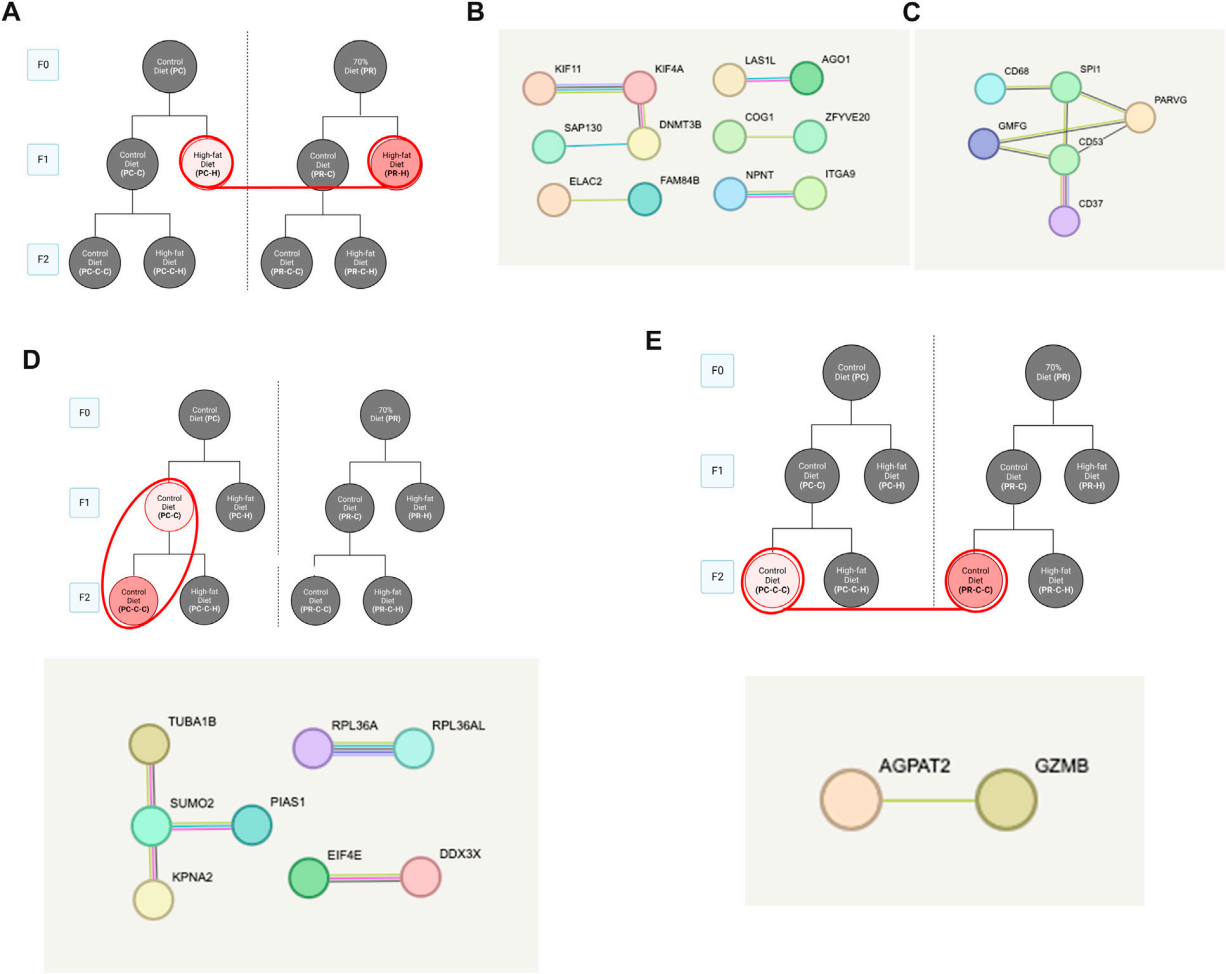
Food restriction comparisons. **(A)** Schematic indicating comparisons for PC-H vs PR-H which revealed (58 DEGs). STRING pathway analysis of **(B)** upregulated and **(C)** downregulated DEGs lacked specific GO term assignments. **(D)** Comparing F1 and F2 offspring with control diet dams (PC-C vs PC-C-C) revealed 45 DEGs with no associated GO terms. **(E)** Comparing effect of F0 food restriction on F2 control diet offspring with (PC-C-C vs PR-C-C) revealed 22 DEGs with no associated GO terms.

Despite the differing degrees of genetic response of each of F1 lipid challenged conditions, comparing these two groups yielded a core group of genes with most bearing functions relating to lipid homeostasis. Other comparisons also produced gene networks but no GO term categories. These groups include F1 high fat conditions up- and downregulated genes (Figures 5A–C), only upregulated genes in F1 *versus* F2 PC arm controls (Figure 5D) and only downregulated genes between the two F2 generation control groups (Figure 5E).

**FIGURE 6:**
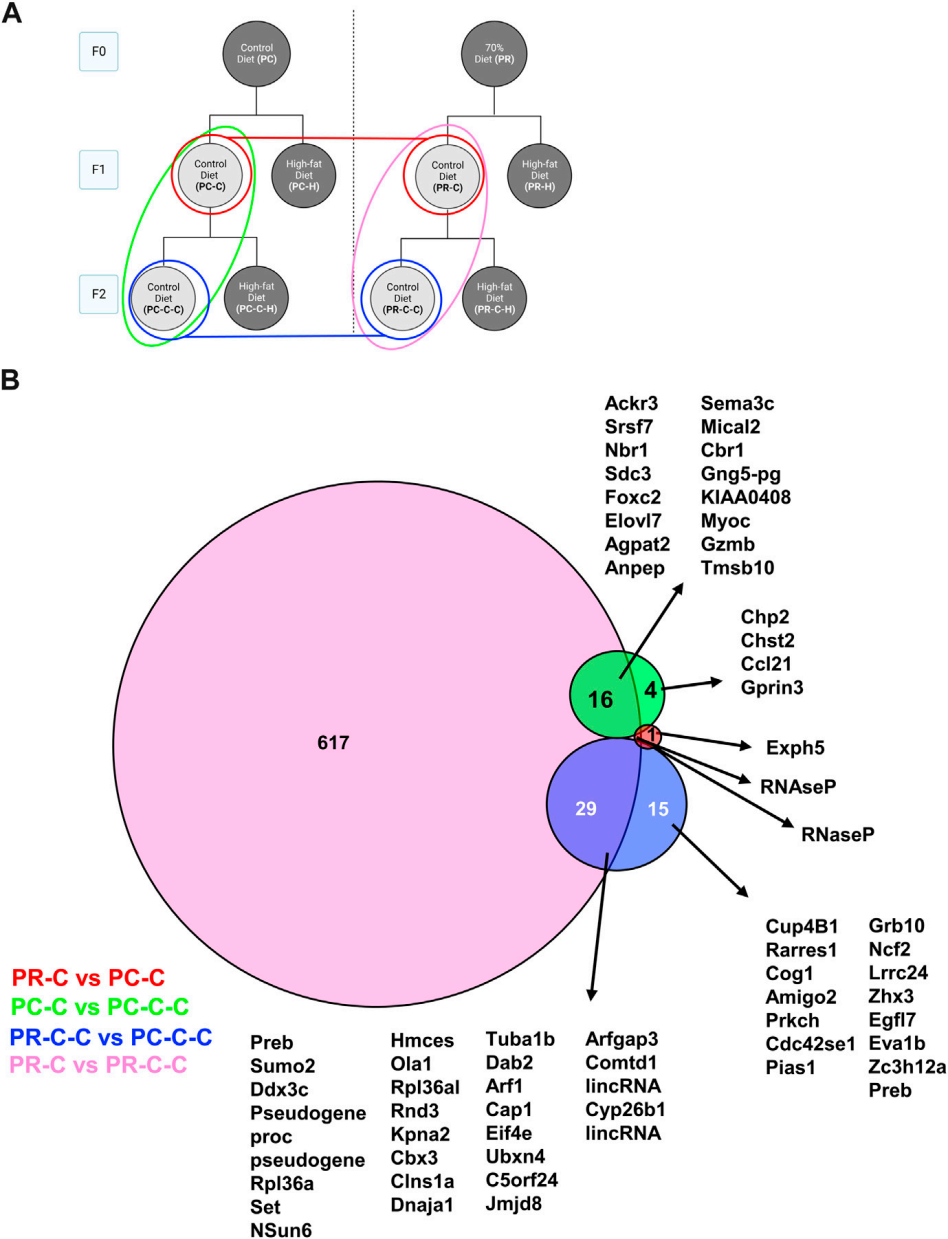
F1 and F2 control diet comparisons with protein network analysis. **(A)** Schematic indicating the comparisons of PC-C vs PR-C (2 DEGs), PC-C vs PC-C-C (45 DEGs), PC-C-C vs PR-C-C (22 DEGs), and PR-C vs PR-C-C (663 DEGs) and the colors of the groupings correspond to the DeepVenn diagram of differentially expressed genes **(B)** Overlapping regions indicate differentially expressed genes shared by two comparator conditions and non-overlapping regions indicate gene uniquely differential for a given comparator group. Genes not listed here are delineated in Supplemental Spreadsheet 1.

To more globally gauge the impact of each dietary challenge, we first compared the differentially expressed genes across all control samples (Figure 6). Only a small number (<30 genes for any group) of transcriptional changes were observed when comparing control-fed PC offspring regardless of generation (i.e., PC-C vs PC-C-C), control-fed F1 offspring (i.e., PC-C vs PR-C), and control-fed F2 offspring (i.e., PC-C-C vs PR-C-C). In contrast, F1 and F2 offspring in the restricted diet F0 arm (PR) of the experiment (i.e., PR-C vs PR-C-C) displayed 617 unique altered genes.

**FIGURE 7:**
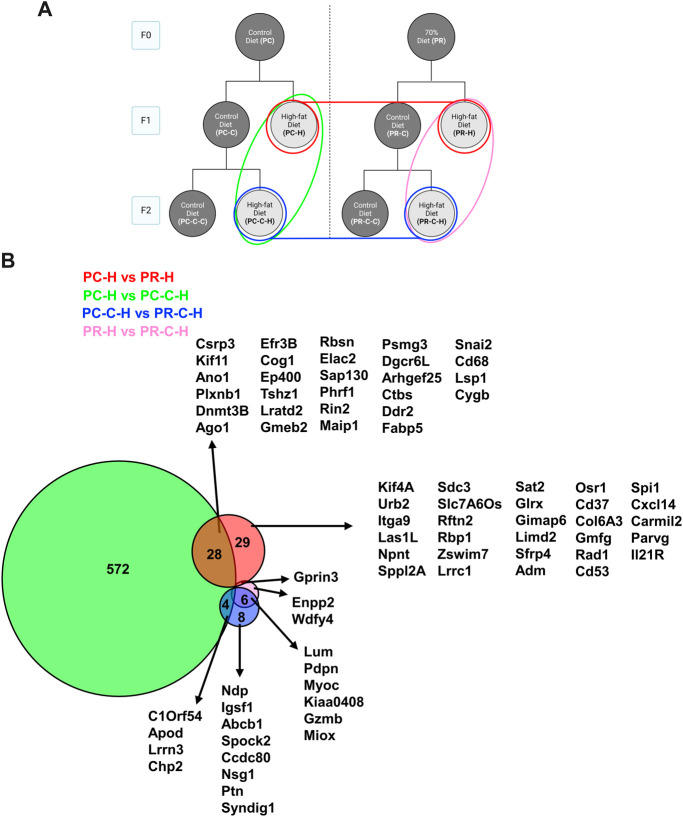
F1 and F2 HFD comparisons with protein network analysis. **(A)** Schematic indicating comparisons of PC-H vs PR-H (16 DEGs), PC-C vs PC-C-C (604 DEGs), PC-C-H vs PR-C-H (18 DEGs), and PR-H vs PR-C-H (9 DEGs) and the colors of the groupings correspond to the DeepVenn diagram of differentially expressed genes. **(B)** Overlapping regions indicate differentially expressed genes shared by two comparator conditions and non-overlapping regions indicate gene uniquely differential for a given comparator group. Genes not listed here are delineated in Supplemental Spreadsheet 1. F1 and F2 HFD comparisons with protein network analysis.

Within the HFD challenged cohorts, the most transgenerational gene expression differences were observed in the comparisons between F2 prenatal controls and their F1 counterparts (i.e., PC-H vs PC-C-H), and the F2 prenatally restricted group (i.e., PC-C-H vs PR-C-H) (Figure 7). The remaining comparisons exhibited only modest gene changes with many shared among conditions (<30 genes for any group).

**FIGURE 8:**
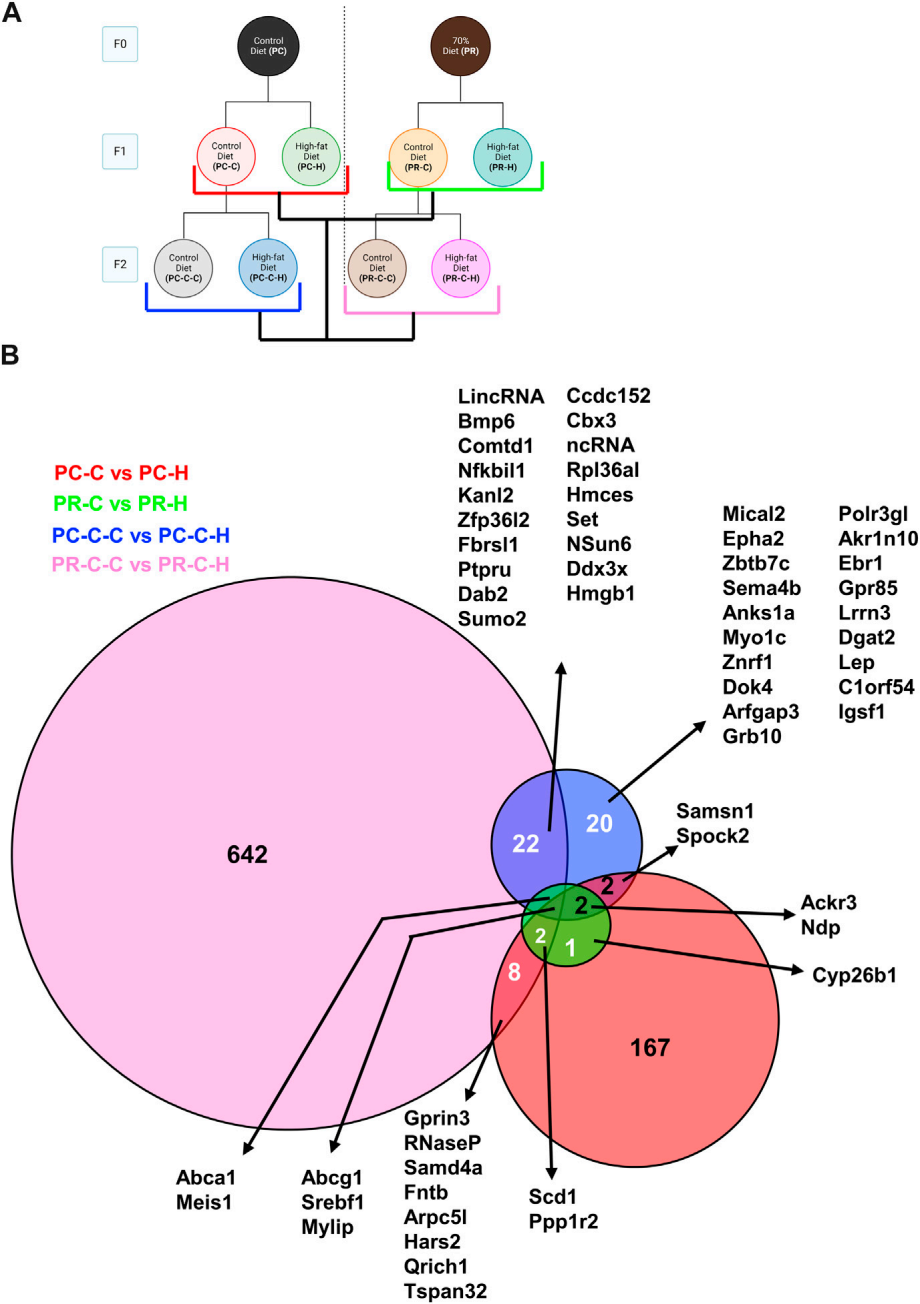
Within generation and transgenerational Control and HFD comparisons with protein network analysis. **(A)** Schematic indicating comparisons of PC-C vs PC-H (185 DEGs), PR-C vs PR-H (16 DEGs), PC-C-C vs PC-C-H (49 DEGs), and PR-C-C vs PR-C-H (679 DEGs) and the colors of the groupings correspond to the DeepVenn diagram of differentially expressed genes. **(B)** Overlapping regions indicate differentially expressed genes shared by two comparator conditions and non-overlapping regions indicate gene uniquely differential for a given comparator group. Genes not listed here are delineated in Supplemental Spreadsheet 1.

Comparisons between prenatal conditions and generations for genes altered between HFD challenge and their respective control group revealed a number of differences (Figure 8). HFD induced a fairly large number of gene changes in the F1 generation from the control F0 arm (167 genes; PC-C vs PC-H) compared to the same challenge in the prenatally restricted diet arm (16 genes; PR-C vs PR-H). This trend appears to reverse in the F2 generation with the control diet arm displaying fewer affected genes (49 genes; PC-C-C vs PC-C-H) while the prenatally restricted diet F2 subjects exhibited many more uniquely altered genes (679 genes; PR-C-C vs PR-C-H). When these groups are compared together, a more complex interaction of shared genes is unveiled with many more affected transcripts being shared by two or more groups (Figure 8B). Of particular interest is the convergence of common genes for all four groups. This includes *Abcg1* (an ATP-binding cassette (ABC) transporter involved in cholesterol efflux (Munch et al., 2012)), *Srebf1* (a basic helix-loop-helix-leucine zipper (bHLH-Zip) transcription factor implicated in regulating sterol metabolism (Nguyen et al., 2021)) and *Mylip* (a E3 ubiquitin ligase known to regulate low-density lipoprotein expression (Emmer et al., 2021)) with all upregulated in high fat challenge, likely representing a coordinated cellular response to a dyslipidemic environment.

## Discussion

We established a transgenerational model to study prenatal food restriction with a second challenge of HFD. We found no differences in birth weight of offspring from the control and food restricted dams. Historically, the Barker group produced the earliest observations indicating an inverse relation between birthweight and mortality from ischemic heart disease (Barker et al., 1989). Since that time, birthweight has been extensively used as a marker for elevated risk of adult-onset chronic disease with many human diseases linked to low birth weight worldwide (Thornburg, 2023). Animal model studies have also shown that malnutrition of various forms can be associated with reduced birthweight of offspring and elevated risks for disease states. However, not all maternal stressors, nutritional or otherwise, lead to a reduction in birthweight. Interestingly, we did not observe a significant decrease in maternal weight or the birth weight in the F1 generation and mirroring other studies of gestational dietary challenges. In the case of humans, the Dutch Hunger winter that spanned the winter of 1943 and spring of 1944 resulted in early gestation undernutrition yet did not result in statistically reduced birthweight (Schulz, 2010). However, studies decades later showed clear associations with chronic conditions including coronary heart disease, obesity, renal dysfunction and type 2 diabetes in this population (Roseboom et al., 2006). Similarly, animal studies have also demonstrated elevated disease risk from nutrition deficit does not necessarily require reduced birthweight (Jones and Friedman, 1982). As Langley-Evans posited (Langley-Evans, 2006), fetal nutrient imbalances can influence lifespan disease risk independent of alter fetal growth. We believe that our observations of clear endothelial transcriptome effects indicate the minimal amount of maternal dietary restriction necessary to engage epigenetic mechanisms have not been established and our data reflects that the activation threshold for transgenerational genomic remodeling is sensitive to more subtle dietary perturbation than has been previously appreciated.

The second challenge of HFD caused a decrease in the rate of weight gain in animals from both prenatal environments (control and food restricted) and both generations (F1 and F2). This is likely due to the animals decrease in total food intake for the HFD. Improvements to this model should include a HFD which is more appealing to the guinea pigs. Importantly, the HFD diet did alter the blood chemistry by increasing cholesterol and decrease triglycerides, similar to other studies with guinea pigs (Fernandez et al., 1992). While there are multiple causes for the increase in cholesterol (*de novo* synthesis of cholesterol, lipoprotein uptake, cholesterol esterification and reverse cholesterol transport) (Lange, 1994) more investigation is needed to determine the mechanism in this model. Hypercholesterolemia is known to cause endothelial cell dysfunction and increase the risk of cardiovascular disease (Feron et al., 1999; Halcox et al., 2009). The molecular mechanisms at work for these cardiovascular phenotypes is only somewhat understood with the cellular basis for transgenerational components of such phenotypes are less clear and this model may provide additional insights. Given that the cardiovascular system is a well-established target of epigenetic reprogramming by maternal dietary restriction (Thornburg et al., 2010; Coppede et al., 2022), we assessed how gene expression changes in the vasculature using the carotid artery, a large, athero-prone vessel, and the unbiased profiling approach of bulk mRNA sequencing. Our acute harvesting of RNA using a guanidinium/phenol flush produced a sample that predominately reflected the endothelial cell transcriptome (Supplementary Figure S3). This is a critical aspect of our analysis as endothelial dysfunction is an underlying component of many cardiovascular diseases (Bonetti et al., 2003; Gimbrone and Garcia-Cardena, 2016). A complete analysis of the observed adaptations in gene alterations is beyond the scope of this report, but the magnitude of effect in the conditions and specific gene alterations merit discussion of their known roles in cardiovascular function and pathophysiology.

Pair-wise comparisons of the F1 generation conditions (Figures 4A–D) produced the surprising finding that offspring from an *ad libitum* diet mother produce a larger number of gene alterations in response to a HFD relative to offspring from a restricted mother (Figure 4). Protein network analysis of the *ad libitum* diet arm of F1 using the STRING algorithm identified several protein networks among both the upregulated (Figure 7B) and the downregulated genes. The upregulated genes were linked to specific Gene Ontology (GO) terms associated with protein folding, consistent with endoplasmic reticulum stress as a driver of endothelial dysfunction (Lenna et al., 2014). Recent reports highlight this effect specifically in metabolic challenges associated with diabetes (Maamoun et al., 2019) and HFD (Zhong et al., 2023). The downregulated genes for PC-C *versus* PC-H exhibited smaller gene networks and produced no GO terms (Figure 7C). Despite the smaller number of affected transcripts, the restricted diet F1 comparison (PR-C *versus* PR-H) produced a number of genes associated with GO terms including: Positive regulation of cholesterol biosynthetic process (*Abcg1*, *Srebf1*), Negative regulation of cholesterol storage (*Abca1*, *Abcg1*), and Regulation of cholesterol biosynthetic process (*Abca1*, *Srebf1*, *Scd1*). Our finding of fewer transcripts associated with the dietary restriction arm of the F1 generation may seem inconsistent with previous reports that indicate a worsening of cardiovascular disease in offspring where maternal dietary restriction occurred (Thompson et al., 2014; Darby et al., 2022). However, it is important to note that our subjects only consumed their HFD for 12 weeks. This duration of HFD was sufficient to alter serum cholesterol and triglycerides (Figure 3), yet it is very possible that our transcriptomes were measured before or after a wave of genomic responses to HFD that underlie observed increases in cardiovascular risk.

Interestingly, a comparison of the affected genes shared by both arms of the F1 generation reveals a shared set of transcripts that include some of the cholesterol genes found in the restricted diet F1 animals (Figure 4E). As described in the results, a number of these genes have been linked to lipid metabolism and in some cases cardiovascular function and disease including: *Abcg1* (Munch et al., 2012), *Srebf1* (Nguyen et al., 2021), *Scd1* (Balatskyi and Dobrzyn, 2023), *Mylip* (Weissglas-Volkov et al., 2011; Emmer et al., 2021), and *Ackr3* (Gencer et al., 2022). A subset of this constellation of transcripts also exists in other conditions within our study as discussed below. The remaining pair-wise comparisons yielded only small relational networks with no ontology terms, which in some cases included both up and downregulated genes (Figures 5A–C), while others yield only upregulated (Figure 5D) or downregulated (Figure 5E) gene networks.

We next extended our analysis to multiple comparisons to determine the common and distinct aspects of endothelial transcriptomes across our cohorts. We first examined how the control groups varied across both F1 and F2 generations (Figure 6A). In comparing the F1 control groups only two differential genes were detected (Figures 6A, B, red), whereas the F2 generation differed by 44 transcripts (Figures 6A, B, blue). The transgenerational differences in each dietary arm yielded distinct numbers of gene differences with the control prenatal diet arm expressing 21 differential genes between F1 and F2 (Figures 6A, B, blue). Alternatively, the restriction prenatal arm controls were found to differ by 663 genes (Figures 6A, B, pink). A number of possibilities exist to explain this large number of differential genes detected between the F1 and F2 restriction controls. Our strict statistical criteria may have filtered these genes in other cohorts despite approaching significance. In some cases, this divergence may reflect the inter-animal variability associated with the fact our animals are outbred. The former option could be verified in future studies by increasing the sample size for each cohort, perhaps through focusing on specific conditions rather than the broad range we chose for our initial characterization. The latter case of genetic diversity poses a more substantive challenge as only a limited number of inbred lines currently exist.

In comparing the HFD cohorts across dietary arms and generations, we also observe one case where large numbers of transcripts differed between conditions (Figure 7). While the F1 HFD cohorts only differ by 58 genes (Figures 7A, B, red), the F2 HFD cohorts by only 18 genes (Figures 7A, B, blue), and the dietary restriction arm F1 and F2 HFD generations only differ by 9 transcripts (Figures 7A, B, pink). In contrast, the control arm F1/F2 comparison produced 604 differential genes between these two *ad libitum* diet control sets. Our comparison of the gene signatures produced by each pair-wise control-diet/HFD set (Figure 8A) revealed that each condition retains a number of unique gene expression patterns, but also has a number of gene sets shared between conditions. It also illuminated the fact that three genes are shared by all four conditions: *Abcg1*, *Srebf1* and *Mylip*. As discussed above, these genes all relate to cardiovascular disease and may represent a wide-spread response of endothelial cells to maintain cellular homeostasis regardless of their epigenetic status. Similarly, an additional 2 genes, *Abca1* and *Meis1*, are shared by all conditions except the F1 control arm comparison. Similar to *Abcg1*, *Abca1* is a cholesterol transporter that may also reflect the increased cholesterol burden associated with HFD. The homeobox transcription factor *Meis1* may represent one of the nuclear factors contributing the observed alterations in gene expression. None of these shared genesets analyses produced GO terms, indicating that these transcriptional alterations may modify diverse pathways within the endothelial cells and will require additional study to determine their individual or synergistic roles in increased cardiovascular risk.

Interestingly, long non-coding RNAs (lncRNAs) were a class of transcripts detected in several of our cohorts. While it has proven difficult to identify *bona fide* orthologs of specific lncRNAs across species, it is clear these genes play crucial roles in the homeostasis of the vascular system (Li et al., 2020; Ding et al., 2022; Tang et al., 2022) and our data would support a role for them in response to dietary perturbations either *in utero* or post-natally. As RNA structural predictions improve, it may be possible to link established lncRNA in mouse or human with those observed in our study.

Taken together, our transcriptomic data indicate that this transgenerational model captures transcriptional differences associated with maternal dietary restriction and also the molecular consequences of a second challenge of HFD, as has been recently demonstrated in another guinea pig model (Sarr et al., 2016). HFD is an important component of this study as it can act to reveal covert alterations in gene regulation which lower the threshold for gene expression changes or only manifest when cells are repeatedly challenged. Other challenges may yield similar or distinct responses primed by prenatal stress, but such associations must await additional transcriptional studies to confirm common regulatory schemes shaping endothelial responses.

## Conclusion

Physiologic parallels are a critical component in identifying appropriate model systems. Our interest is in determining molecular mechanisms that contribute to the transgenerational effects of fetal epigenetic remodeling. Here we present data from our transgenerational model applying maternal food restriction and determining how it alters genomic conditioning based on transcriptional responses to a second challenge such as HFD in our case. Thoughtful selection of the guinea pig based on its hemochorial placenta, precocious organ development timeline, and postnatal lipid profiles make this model translationally relevant to study the endothelial underpinnings of developmental programming. Pregnancy rates and litter sizes were unchanged by maternal undernutrition, and female F1 offspring also had comparable pregnancy rates and litter sizes making this a viable transgenerational model. Our transcriptional analyses demonstrate that this model system reproducibly undergoes genetic remodeling in response to dietary challenges both pre- and postnatal. It also illuminates gene sets that are potentially primed by familial history and others that exhibit responsiveness to these insults regardless of history. One important factor that our sample size precluded is the effect of sex on the response to either maternal stress or dietary challenge, but this component will be a critical aspect for future studies. The determination of how generalizability these finding are will require also additional studies that track transcriptional responses to other physiologic challenges secondary to material diet augmentation and comparison with our data. Here, we utilized HFD to as a second insult and measured alterations in endothelial transcriptional responses between dietary conditions. Future comparisons using the paradigm described here with other secondary insults such as cigarette smoke, hypertension or diabetes will provide additional insights into how the epigenetic effects of maternal diet permeate future generations.

## Limitations of the study

The limitations associated with the current study include at least seven aspects. First, the relatively small study size due to the large number of treatment groups used. Statistical power would be increased by increasing animal numbers in future studies. Second, we lack information regarding the prenatal environment of the original F0 generation females that may differ significantly and introduce additional variability. Third, the guinea pigs used in this study were not congenic and thus inter-individual genetic difference may also contribute to any observed variation. Fourth, future studies will also need to further optimize the HFD dietary modifications to minimize the initial weight loss we observed. Fifth, our focus on the carotid artery limits the conclusions that can be drawn regarding other vascular beds, including resistance arteries. Sixth, animal numbers limiting our ability to assess sex-dependent effects that have been noted previous in the literature regarding maternal dietary effects. Finally, we do not have *in vivo* or *ex vivo* measures of vascular function with which to correlate our findings.

## Data Availability

The data presented in the study are deposited in the NCBI Gene Expression Omnibus (GEO) repository, accession number GSE244302.

## Supplementary material

Supplementary Figure S1
**(A)** Total serum cholesterol and **(B)** triglyceride levels after 3 weeks of 70% food restriction in non-pregnant guinea pigs. At week 0 and 3, and maternal control diet had *n* = 4 and maternal food restriction had *n* = 4. All data points are displayed and analyzed using Wilcoxon Test. Data were checked for normality using a Shapiro-Wilk test and quantile-quantile plot as well as equality of variance using Levene’s Test. **p* < 0.05.

Supplementary Figure S2 Dam body weight during pregnancy and lactation. Repeated measures ANOVA did not show significant difference in body weight during pregnancy and lactation between all groups of F0 control diet (*n* = 4), F0 restricted diet (*n* = 4), F1 from the prenatal control diet F0 dam (*n* = 2), F1 from the prenatal restricted F0 dam (*n* = 4). All data points are displayed as mean ± SD.

Supplementary Figure S3 Cell Enrichment. Averages of gene counts were calculated for each group (*n* = 3) except PC-C-H which has *n* = 2). Samples were confirmed for endothelial cell enrichment using cell markers *PECAM1*, *CDH5*, and *TIE1*.

Supplementary Table S1 F1 and F2 offspring kilocalorie/kg/day intake before (“baseline”) and at the conclusion of (“After Diet”) the postnatal high fat diet challenge. F1 HFD offspring had significantly higher kilocalories/kg/day from fat. Food restricted diets had significantly less total kilocalorie/kg/day intake including kilocalorie/kg/day from fat, protein and carbohydrates. For F1 prenatal control diet offspring, *n* = 3 for postnatal control diet (PC-C) and *n* = 4 for high fat postnatal diet (PC-H). For F1 prenatal food restricted diet offspring, *n* = 3 for postnatal control diet (PR-C) and *n* = 4 for high fat postnatal diet (PR-H). For F2 prenatal control diet offspring, *n* = 3 for both postnatal control (PC-C-C) and high fat diet (PC-C-H). For F2 prenatal food restricted diet offspring, *n* = 3 for postnatal control diet (PR-C-C) and *n* = 7 for high fat postnatal diet (PR-C-H). All data points are displayed as mean ± SD and were checked for normality using a Shapiro-Wilk test and quantile-quantile plat as well as homogeneity of variance using Levene’s Test. Data were analyzed using Kruskal-Wallis Test with Dunn’s post-hoc test using Bonferroni’s correction.

Supplementary Datasheet S1 Contains animal information (Tab 1), normalized count matrix for each individual sample (Tab 2) and differential genes (subsequent tabs) based on pair-wise comparisons of conditions as described in the text. Genes indicated in green were manually curated as the automated gene assignments for the guinea pig genome are incomplete in some cases.
