# Supplementary material for: Development of a novel Guinea Pig model producing transgenerational endothelial transcriptional changes driven by maternal food restriction and a second metabolic insult of high fat diet
Source: Front Physiol. 2023 Oct 24;14:1266444. doi: 10.3389/fphys.2023.1266444 (PMC10628814; doi:10.3389/fphys.2023.1266444)
Supplement: Supplementary file 1 [file Table1.pdf]

Supplemental Table 1

	Total kcal/kg/day		Fat kcal/kg/day		Protein kcal/kg/day		Carbohydrate kcal/kg/day	
**F1**	**Baseline**	**After Diet**	**Baseline**	**After Diet**	**Baseline**	**After Diet**	**Baseline**	**After Diet**
PC-C	241 ± 62^{A}	222 ± 72^{A}	32 ± 8^{A}	30 ± 7^{A}	64 ± 17^{AB}	59 ± 19^{A}	145 ± 37^{AB}	134 ± 43^{A}
PC-H	247 ± 61^{AB}	165 ± 82^{B}	40 ± 17^{BC}	62 ± 31^{B}	64 ± 17^{A}	34 ± 17^{B}	135 ± 48^{C}	67 ± 33^{B}
PR-C	293 ± 80^{C}	241 ± 105^{A}	39 ± 11^{BD}	32 ± 14^{A}	78 ± 21^{C}	64 ± 28^{A}	176 ± 48^{A}	145 ± 63^{A}
PR-H	262 ± 79^{B}	209 ± 97^{C}	42 ± 21^{CD}	77 ± 35^{C}	68 ± 21^{BC}	43 ± 20^{C}	141 ± 58^{BC}	82 ± 37^{C}
**F2**								
PC-C-C	235 ± 57^{A}	196 ± 59^{ABC}	31 ± 8^{A}	26 ± 8^{A}	62 ± 15^{A}	52 ± 16^{A}	142 ± 34^{A}	118 ± 35^{A}
PC-C-H	247 ± 76^{A}	181 ± 79^{AD}	41 ± 23^{B}	60 ± 27^{B}	64 ± 20^{A}	39 ± 17^{B}	130 ± 53^{A}	61 ± 28^{B}
PR-C-C	240 ± 78^{A}	213 ± 76^{B}	32 ± 10^{AB}	28 ± 10^{A}	64 ± 21^{A}	57 ± 20^{A}	144 ± 47^{A}	128 ± 45^{A}
PR-C-H	241 ± 85^{A}	194 ± 97^{CD}	37 ± 21^{AB}	64 ± 31^{B}	63 ± 22^{A}	42 ± 22^{B}	132 ± 53^{A}	68 ± 35^{B}
